# A New Clinical Section in *PLoS Neglected Tropical Diseases*

**DOI:** 10.1371/journal.pntd.0000760

**Published:** 2010-09-28

**Authors:** Carlos Franco-Paredes, Albert I. Ko, Serap Aksoy, Peter J. Hotez

**Affiliations:** 1 Hospital Infantil de Mexico, Federico Gomez, Mexico, D.F., Mexico; 2 Department of Medicine, Emory University, Atlanta, Georgia, United States of America; 3 Oswaldo Cruz Foundation, Rio de Janeiro, Brazil; 4 Weill Medical College of Cornell University, New York, New York, United States of America; 5 Yale School of Public Health, New Haven, Connecticut, United States of America; 6 Department of Microbiology, Immunology, and Tropical Medicine, The George Washington University, Washington, D.C., United States of America; 7 Sabin Vaccine Institute, Washington, D.C., United States of America

*PLoS Neglected Tropical Diseases* (*PLoS NTDs*) provides a platform of information that links multiple scientific disciplines in the battle against the substantial public health and economic impact imposed by neglected tropical diseases (NTDs). The types of articles published in *PLoS NTDs* include health policy; control, elimination, and eradication programs; epidemiological and operational research; basic pathobiology, translational research, and development; development of new diagnostic tests; and many other areas of action.

*PLoS NTDs* also reaches health care practitioners caring for individuals suffering from NTDs through the clinical symposium section. To effectively target this audience, the clinical symposium section delivers critical information in the case management of NTDs backed by the expertise and commitment of our clinician editors. Up until now, the clinical symposium section has included two types of article formats: a case-report format, and case-based learning articles that consist of a question and answer format illustrating key clinical issues in the diagnosis and management of NTDs. Both types of articles are presented in a simple format that makes them easy to apply to direct patient care with an emphasis on key learning points to provide clinical pearls or take-home points.

In this issue of *PLoS NTDs*, we are pleased to launch a new section to complement the clinical symposium section with the publication of the first Photo Quiz. Our first Photo Quiz case addresses the diagnosis and management of actinomycetoma in both resource-rich and resource-poor settings [1]. This new clinical section is devoted to question-and-answer challenges through the use of specific NTD clinical images, each illustrating a key clinical finding in the diagnosis, management, or prevention of a selected disease.

We encourage the audience of clinical practitioners to submit relevant clinical, radiographic, or microbiological images. Some examples may include images of a clinical sign characteristic of infection with a particular pathogen; radiographic images that may be identified in patients suffering from a particular NTD; electrocardiograms or echocardiograms with relevant abnormalities; or a video of the patient's clinical signs. From a laboratory/pathology perspective, we also encourage submission of pathogens identified in clinical specimens. All images will be published under the Creative Commons Attribution License (http://www.plosntds.org/static/license.action), followed by discussion of key learning points. Upon submission of a manuscript, cases will be assessed by the editorial office and subsequently undergo a formal peer review process following the *PLoS NTDs* editorial review policy (http://www.plosntds.org/static/guidelines.action#overview). Submissions for the Photo Quiz section should follow the two-part format outlined in Box 1.

**Box 1. Instructions for Photo Quiz Submissions**

**A. Case Discussion and Question**

- The submitter(s) should provide an initial brief presentation of a clinical case (history, physical examination, relevant laboratories, etc.) with key clinical images, which should invite a diagnosis from the reader by posing a single-sentence question about the case.
- The case presentation and question should be written in a single paragraph and should be accompanied by no more than 2 images/figures (no more than 150 words).
- Similar to the Symposium manuscripts, authors must obtain written consent from the patient using our consent form (also available in French, Portuguese, and Spanish). Consent forms are available at http://www.plosntds.org/static/guidelines.action#supporting.

**B. Answer(s)/Discussion**

- The answer should give the diagnosis followed by a discussion of the most relevant clinical issues (no more than 1,200 words).

**C. Key Learning Points**

- Authors must include a box that lists 3–5 key learning points of the case similar to other clinical sections of *PLoS NTDs*.

**D. References**

- No more than 10 references.
